# Supplementary material for: Synergistic Crosstalk of PACAP and Notch Signaling Pathways in Bone Development
Source: Int J Mol Sci. 2025 May 26;26(11):5088. doi: 10.3390/ijms26115088 (PMC12154205; doi:10.3390/ijms26115088)
Supplement: Supplementary file 1 [file ijms-26-05088-s001.zip › ijms-3606207-supplementary.pdf]

Synergistic crosstalk of PACAP and Notch signalling pathways in bone development

Vince Szegeczki [a#], Andrea Pálfi [a#], Csaba Fillér[a], Barbara Hinnah[a], Anna Tóth[a], Lili Sarolta Kovács[a], Adél Jüngling[b], Róza Zákány[a], Dóra Reglődi[b], Tamás Juhász[a]*

Supplementary Figures

Negative controls of NFATc1 immunohistochemistry

To help validate the specificity and reliability of antibody staining and to ensure that any observed signals are due to specific binding rather than non-specific interactions or background noise, negative controls were done in immunohistochemistry. Omitting the primary antibody entirely served as a negative control to identify background staining caused by secondary antibodies or other reagents.

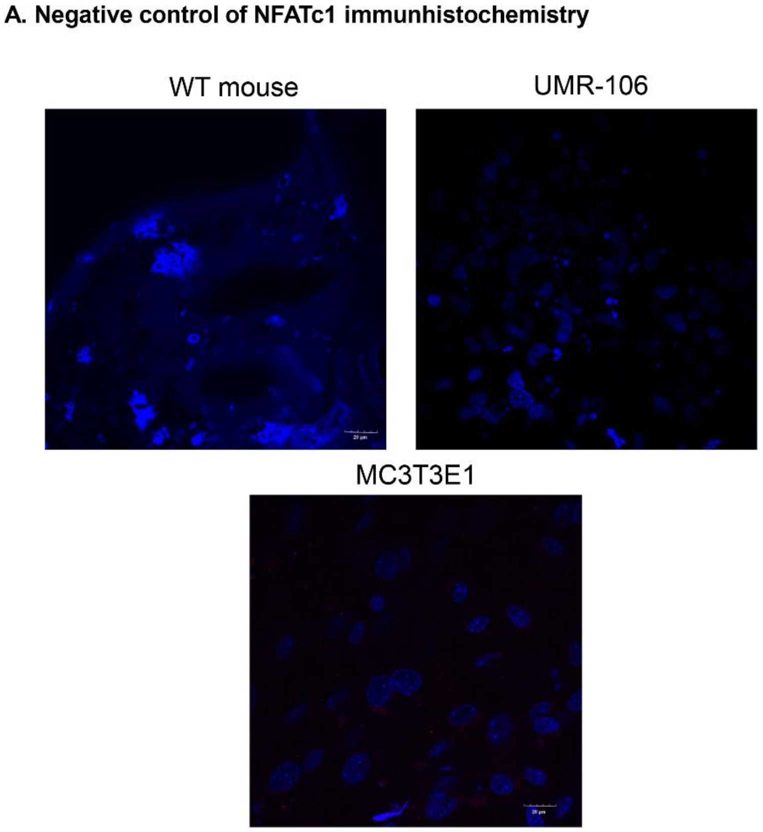

Supplementary Figure 1. Negative controls of immunocytochemistry of NFATc1 in femurs of WT and PACAP KO mice, in UMR-106 and MC3T3E1 cells. Original magnification was 60×. Scale bar, 20 µm. Shown are representative results of 3 independent experiments.
